# Profile of Voice Professionals Seen in a Tertiary Health Center

**DOI:** 10.1016/S1808-8694(15)31118-6

**Published:** 2015-10-20

**Authors:** Felipe Sartor Guimarães Fortes, Rui Imamura, Domingos Hiroshi Tsuji, Luiz Ubirajara Sennes

**Affiliations:** 1 Ex-resident and physician. Teacher and collaborator at the Otorhinolaryngological Clinical Division of the HCFMUSP, Clinical Hospital of the Sao Paulo University Medical School (HCFMUSP); 2 Doctor in Otorhinolaryngology at the FMUSP. Assistant physician of the Otorhinolaryngological Clinical Division of the HCFMUSP; 3 Lecturer and Professor from the FMUSP. Head of the voice clinic (Grupo de Voz) of the HCFMUSP; 4 Lecturer and Professor from the FMUSP. Associate Professor of Otorhinolaryngology at FMUSP. Head of the Buccopharyngolaryngology Unit at HCFMUSP

**Keywords:** voice professional, prevention, epidemiology, work related laryngopathy

## Abstract

**Summary:**

Work-related laryngopathy may have negative consequences for voice professionals.

**Aim:**

To analyze the profile of voice professionals seen in a tertiary level hospital.

**Study design:**

a longitudinal historical cohort.

**Methods:**

A retrospective analysis of patient files. Diagnosis was reached using videostroboscopy.

**Results:**

163 patients (119 females and 44 males) were seen. The mean age was 36.5 years. Professionals included spoken voice users (salesman, teachers, telemarketers, receptionists, health professionals) and singers. The most frequent diagnoses were: minor structural changes (33%), nodules (22%), Reinke's edema (10%), and polyps (6%). A correlation was observed between smoking, age and gender; there was an association between smoking and Reinke's edema, leucoplasia and tabagism, females and Reinke's edema, nodules and minor structural changes, and also between patients aged over 40 years and Reinke's edema, and patients under 40 with nodules, laryngitis, and minor structural changes. Symptoms lasted more than 6 months in 74% of patients.

**Conclusion:**

The profile of voice professionals seen in a tertiary hospital included spoken voice patients and singers. In our study minor structural changes predominated, followed by nodules, Reinke edema and polyps.

## INTRODUCTION

Voice is an essential tool in the lives of many professionals. Roughly 25% of the economically active population consider voice as a fundamental working tool.^1^,^2^ Dysphonia, little valued until recently, today is considered a significant disturbance, with consequences that directly influence the professional and social life of an individual.^3^

Titze et al.^4^ and Fritzell^5^ reported that professionals with the highest risk of having voice problems are singers, followed by consultants, teachers, lawyers, pastors, telemarketers, salespersons, and health professionals. An important point is that a professional voice user will seek medical help only if he or she is aware of its importance, among other things.

The aim of our study was to survey professional voice users seen at a voice clinic (Grupo de Voz) in a tertiary health center, and to characterize those professionals that seek the outpatient clinic with greater frequency, as well as the incidence of diseases in these persons.

## MATERIAL AND METHODS

We conducted a cross-sectional retrospective study to review the files of patients seen at the voice clinic in a tertiary health center between 1990 and 2003. Only professionals that considered their voices as an essential working tool, that sought medical help due to voice complaints, and that had complete medical documentation including videolaryngostroboscopy, were included in the study. This study was approved by the Research Ethic Committee of the FMUSP Otorhinolaryngology department.

Patients were divided into 9 categories according to their profession: teachers, actors, singers, salespersons, telephone operators, telemarketers, receptionists/secretaries, missionaries, health professionals, and miscellaneous (judges, lawyers, tourist guides and publicity professionals). The diagnosis for each patient was made by using videolaryngostroboscopy, which was analyzed by the same medical team, and divided into the following categories: nodules, polyps, minor structural changes,^6^ Reinke's edema, papilloma, leukoplasia, tumor, vocal fold palsy, functional disturbance, neurological disease, and laryngitis due to laryngopharyngeal reflux (LPR). This last diagnosis was made based on clinical and laryngoscopic findings (edema, hyperemia, pachydermia on the posterior cricoid, interarytenoid region, and arytenoids). Duration of dysphonia (taken as the time up to the moment of evaluation at the voice clinic), age, gender and smoking habit were also annotated.

Patients with a history of surgery, radiotherapy or head and neck neoplasms were excluded.

Patients were classified according to the general distribution of professionals and diagnoses, the duration of the complaint and the proportion of diagnoses relative to profession, gender, age group (40 years or less, and over 40 years), and association with smoking.

We used the software SPSS (Statistical Package for Social Sciences), version 10.0, for statistical analysis, adopting a 5% (α=0.05) significance level for statistical tests. We also used the Chi-squared test for the analysis of proportions.

## RESULTS

Our study included 163 patients, 119 women (73%) and 44 men (27%). Age varied from 16 to 72 years, the mean age was 36.5 years with a standard deviation of 12.1 years. Patients were divided into two groups according to age (40 years or less, and over 40 years), with 102 patients (63%) in the first group, and 61 patients (37%) in the second group. There were 105 non-smokers (65.2%), and 56 (34.8%) smokers.

**Chart 1:**
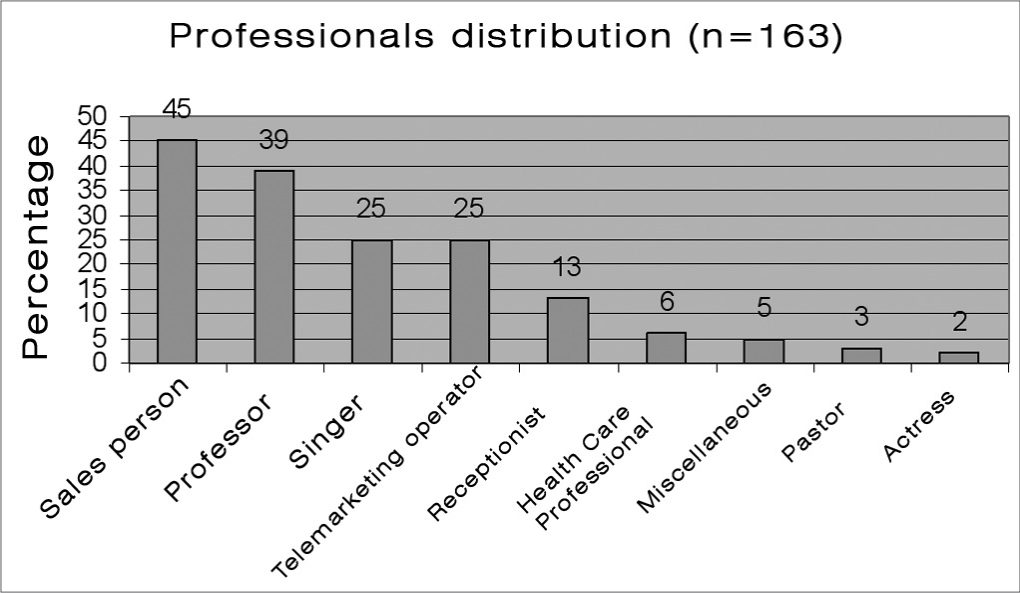
Professional voice users seen at the HCFMUSP voice clinic.

**Chart 2:**
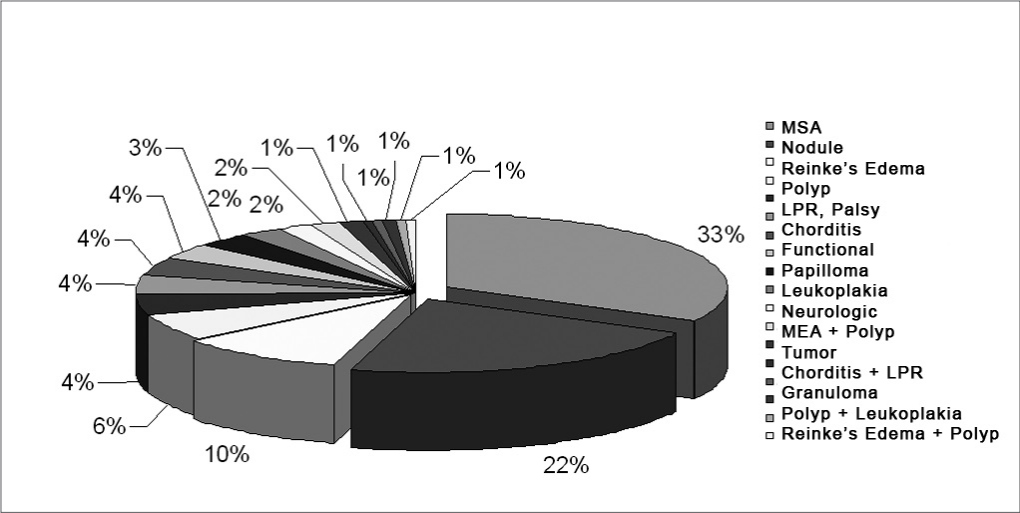
Distribution of diagnoses of professional voice users seen at the voice clinic.

Chart 1 shows the distribution of patients according to profession, with their relative percentages. Chart 2 shows the distribution of patients according to the diagnosis. Within the group 'minor structural changes' we included: vocal cord sulcus, epidermoid cyst, mucosal bridge, laryngeal microdiaphragm and vascular dysgenesia.^6^

**Chart 3:**
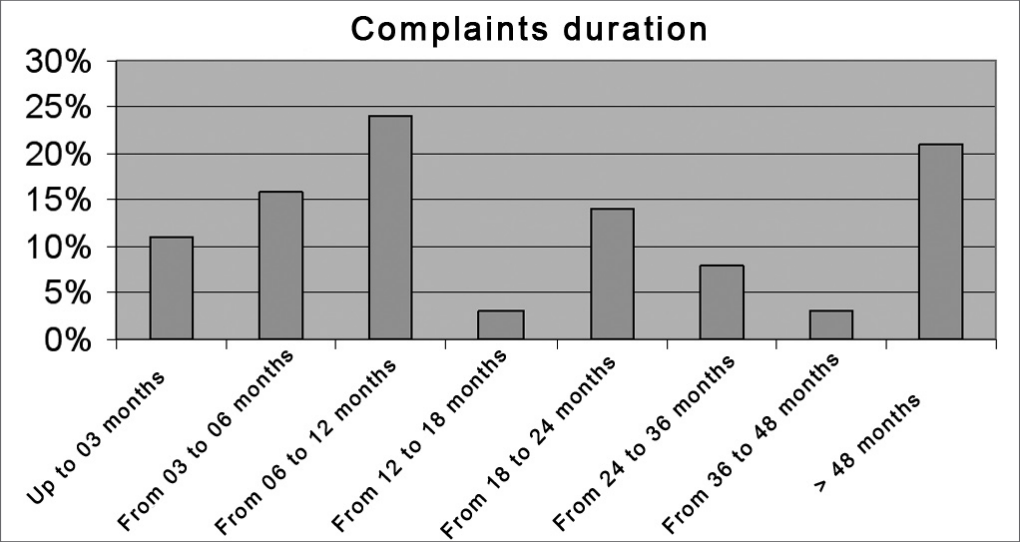
Progression time of a complaint among professional voice users seen at the voice clinic.

Duration of symptoms until assessment at the voice clinic was one year or less in 50% of patients, and up to two years in roughly 70% of patients (Chart 3).

A statistically significant correlation was seen between diagnoses and sex (p=0.004), age (p<0.001) and sex (p=0.002), with a correlation between women and minor structural changes, nodules, and Reinke's edema. Other correlations were smoking with Reinke's edema and leukoplasia; Reinke's edema with age over 40 years; and younger age with nodules, corditis and minor structural changes.

The distribution of diagnoses according to the most prevalent professions in this study is shown on Table 1. However, due to the limited number of cases, we were unable to analyze the statistical significance of this difference.

**Table 1:** Distribution of the most frequent diagnoses in professional voice users and professional singers.

	Voice		Singing	
	Salespersons	Teachers	Telemarketing	Singers
Minor structural changes	30%	32%	40%	24%
Nodules	10%	25%	24%	36%
Reinke's edema	12%	16%	8%	4%
Polyps	10%	13%	4%	8%
Corditis	4%	5%	-	12%
OTHERS	LPR 12%	functional 3%	functional 8%	LPR 12%

## DISCUSSION

Communication has acquired a growing importance in the labor market, particularly for those professionals that depend on it as their main working tool.^3^,^7^ Example of such professionals include teachers, actors, singers, receptionists, telemarketers, lawyers, pastors, health professionals, and others. For these professionals, dysphonia can render them incapable of practicing their profession, resulting in absence from work, reduced productivity, and even the need to change profession.^3^,^8^ Studies with telemarketers, for instance, have revealed that occupational dysphonia may lead to diminished sales and a direct impact on productivity.^2^

Dysphonia may result from an interaction between hereditary, behavioral, lifestyle, and occupational factors. Various studies have related occupational activity with dysphonia, where it is believed that the main causal factor is excessive voice use, causing vocal fold trauma, with laryngitis being the most common finding.^2^,^8^ However, one should add a variety of environmental factors that may be indirectly related to work and that can contribute to the problem, such as exposure to chemical irritants (formaldehyde, chrome, mercury, sulfuric acid), inadequate temperature and humidity levels, background noise, and poor acoustics (these are reported mainly by teachers, actors, singers and telemarketers).^1^,^13^ Furthermore, limited recovery time and stress are also considered risk factors for occupational laryngopathy.^3^

Lifestyle habits may also be harmful to the voice. Among them we may mention smoking, alcohol abuse, and laryngopharyngeal reflux (frequently related to inadequate eating habits and behaviors).^7^ There was a high prevalence of smokers in our sample, which showed a correlation with Reinke's edema and leukoplasia. Smoking can cause vocal fold edema, inflammation,^7^ and altered mucus; it has been observed that smokers have a lowered fundamental voice frequency.^12^ Adding to this, alcohol and an inadequate diet are related to LPR.

Reflux laryngitis is associated with increased abdominal pressure on phonation and anxiety, where morning halitosis, dry mouth, sensation of a foreign body in the throat, hoarseness, and a need for extra vocal warming up time are the most common symptoms.^7^,^9^, ^10^ In our study we observed laryngeal alterations suggesting reflux, especially associated with nodules and minor structural changes.

Results of two studies,^4^,^5^ in which the prevalence of various professional categories were seen in voice clinics, allowed Williams1 to stratify the risk of dysphonia according to the professional activity. Professions at higher risk were: professional singers (singers), followed by professional voice users (receptionists, teachers, telemarketers, pastors, lawyers, tourist agents and health professionals).5 In our sample, the most frequent groups were: salespersons (27.6%), teachers (23.9%), singers (15%), telemarketers (15%), receptionists (8%), health professionals (3.7%), with actors, pastors and others at lower percentages. We would like to point out that our sample was composed of patients seen at a public hospital, therefore subject to a social selection bias. Furthermore, patients seek medical help when aware of the problem and the importance of his or her voice.

Laryngitis (or corditis) is reported as the most common finding in work-related dysphonia.7,8 In our series, corditis was diagnosed in only 4% of cases. This difference may be explained by the fact that we studied patients who sought the hospital for the treatment of vocal problems, many of them seeking medical help probably due to the persistence of symptoms. Looking at the mean symptom onset time, we observed that most of the patients had a chronic condition (structural vocal fold change or mucosal lesions). Acute problems were less frequent, and usually resolved spontaneously, such as cases of corditis.

We saw a higher prevalence of minor structural changes compared to nodules, which disagrees with other findings in literature.^14^ This difference may be related to the natural progression of these entities: nodules are lesions that generally improve with speech therapy and voice rest, while minor structural changes are chronic conditions, which usually to not resolve spontaneously or without medical treatment.^15^ Thus, the high prevalence of minor structural changes (33%) is probably the result of our voice clinic attending patients referred to a tertiary medical center after having been screened and possibly treated first in other health units.

Minor structural changes are lesions that alter the structure of the cord mucosa, involving the epithelium and the surface layer of the lamina propria.^6^ The etiology is still being debated, and although some authors consider these changes as being traumatic,15 other authors believe they are congenital lesions. Thus, dysphonia is a common finding in relatively young patients. We found an association between minor structural changes, age below 40 years and the female gender.

There was an association between vocal nodules, age below 40 years and women, similar to other published papers in medical literature for the general population. Nodules are associated with incorrect voice use.^15^

Reinke's edema is mostly related with smoking and to a lesser degree with voice abuse.7,15 We found a significant association between Reinke's edema, smoking, women, and age over 40 years.

Polyps predominate in men between ages 30 and 45 years, and are related to vocal cord trauma.^15^ It may also be related to minor structural changes in up to 20% of cases.^16^ Our series did not show this association frequency, which may be higher after direct laryngoscopy.

Considering the professional groups separately, literature shows that up to 80% of teachers have voice complaints.^14^,^17^, ^18^ Visible lesions on laryngoscopy are found in up to 20% of cases. Among the most frequent conditions found by nasofibrolaryngoscopy we found nodules (43%), Reinke's edema (17%), hypertrophy of bands (12%), polyps (8.7%), and cysts (4.2%).^14^ The main diagnoses we found in teachers were minor structural changes and nodules. As mentioned before, not only our patients are a biased sample (professionals seen at a tertiary public hospital, after screening, with a predominance of chronic cases), our diagnoses in this study were made by rigid telescopic laryngoscopy (70°) and videostroboscopy, which increased diagnostic sensitivity. Studies have shown that videostroboscopy may change the first diagnosis in up to 18% of patients, providing additional diagnostic information in up to 29% of cases. Its use may therefore bring important information in the assessment of professional voice users.^19^ Another factor which may have contributed to this finding is that for many professional voice users, vocal resistance - the ability to use voice during prolonged periods - is more important than voice quality itself, which on the other hand is essential for singers and actors. Many of these professionals do not seek medical help except when dysphonia affects their performance. Minor structural changes, such as vocal fold structural lesions, tend to worsen the voice and produce fluctuations of dysphonia, progressing with excessive use.

According to literature data, up to 44% of singers have dysphonia,^7^,^9^ with inflammatory lesions being the most common finding (27%).^7^ The main cause of the lesion is excessive use of the voice and tension on the vocal folds, usually a consequence of incorrect compensation following (commonly viral) laryngitis.^8^ A further factor is the lifestyle of many of these professionals (smoking, inadequate diet with an increased risk of GERD), which has an important role on the genesis of vocal changes.^9^,^11^ In our series the main diagnosis in singers was nodules, followed by minor structural changes and Reinke's edema. There was a higher prevalence of nodules in professional voice users (teachers, salespersons and telemarketers) in our series compared to minor structural changes. This difference may be explained by the possibility of voice abuse associated with singing, particularly if the technique is inadequate, and also by the probable natural selection of congenital minor structural changes in this population.

Delays in making a correct diagnosis in these professionals may result in incorrect treatment, causing or worsening laryngeal lesions, increased absenteeism from work, possibly compromising their career.^3^,^8^ In our study about 70% of patients had voice symptoms lasting up to two years (11% up to 3 months, 16% between 3 and 6 months, and 41% between 6 months and 2 years), and they still remained with no precise diagnosis. Many had left their activities or had limitations to perform professionally.

Professional voice users find it difficult to leave their daily activities. When deciding to recommend rest for these professionals, the possibility of vocal fold lesions becoming even worse with its accompanying risks should be evaluated.^8^ Prevention programs are important, and should focus on helping professionals to become aware of their problems, to recognize early symptoms (voice fatigue, dry cough, and changes in voice pitch), and also to practice adequate vocal hygiene. This includes good hydration, avoidance of irritants such as active or passive smoking, avoidance of dust, an adequate diet with LPR control, and rest in cases of upper airway infection.^1^,^7^,^8^

## CONCLUSION

The patients that were referred to the voice clinic (Grupo de Voz) of a tertiary health center included both professional voice users (salespersons, teachers, telephone operators, and telemarketers) and vocal performers (singers). We observed a predominance of minor structural changes, followed by nodules, Reinke's edema and polyps, in that order.
